# Surgical Approach and Further Management of Intracranial Hemangioendothelioma With Double Location: A Case Report

**DOI:** 10.7759/cureus.32072

**Published:** 2022-11-30

**Authors:** Alan Hernández-Hernández, Eliezer Villanueva-Castro, Pedro Leonardo Villanueva-Solórzano, Javier Degollado-García, Gerardo Cano-Velázquez, Martha Lilia Tena-Suck, Miguel Angel Ramos-Peek

**Affiliations:** 1 Department of Neurosurgery, National Institute of Neurology and Neurosurgery Manuel Velasco Suárez, Mexico City, MEX; 2 Department of Neuropathology, National Institute of Neurology and Neurosurgery Manuel Velasco Suárez, Mexico City, MEX

**Keywords:** double location, cranioplasty, surgical resection, intracranial, hemangioendothelioma

## Abstract

Hemangioendotheliomas are highly vascularized lesions, and their intracranial presentation is extremely rare. We present the case of a 65-year-old female patient who was evaluated for cranial deformity, headache, and left hemiplegia. Two bone lesions that were destroying and expanding the bone diploe with intracranial extension were identified in the fronto-temporal and parietal regions. Both lesions were multilobed and showed heterogeneous behavior. Mixed hemangioendotheliomas were identified after the successful resection of both tumors in two separate surgical procedures. The prognosis of this type of tumor with an intracranial location is not well-defined because there are too few reported cases.

## Introduction

Hemangioendotheliomas (HE) were first reported in 1982 and were characterized as tumors with a vascular component and an epithelioid appearance that usually occur in soft tissues [1]. They can be found in the lungs, liver, lymph nodes, and heart [1,2]. HE is classified into five types: epithelioid, with spindle cells, papillary, kaposiform, and, more recently, the one with mixed components [1,3]. Intracranial HE appearance is extremely rare, representing only 0.02% of all brain tumors, and they can originate from the dura mater, brain, or skull bone [4-7]. Here, we present a case of mixed hemangioendothelioma with multiple locations and invasion of bone and soft tissue.

## Case presentation

Medical history and physical examination

We present a 65-year-old woman who has had systemic arterial hypertension for 20 years. She first presented a head lump in the right frontal zone 12 years earlier without receiving any medical treatment, then two other lumps, in the parietal and temporal zones on the same side, appeared after 11 years. Then she started suffering from mild headaches, gait disturbances, left hemiparesis, sensitive loss on the left side of the body, urinary incontinence, and left hemineglect for a year, for which she self-prescribed pain medication without improvement until she was referred to our hospital by her family physician. Physical examination revealed frontal, parietal, and temporal palpable masses greater than 7 cm in diameter, left lower visual field defects, left central facial palsy, and a paretic left gait. There was no evidence of meningism or impaired higher mental functioning.

Diagnostic tests

**Figure 1:**
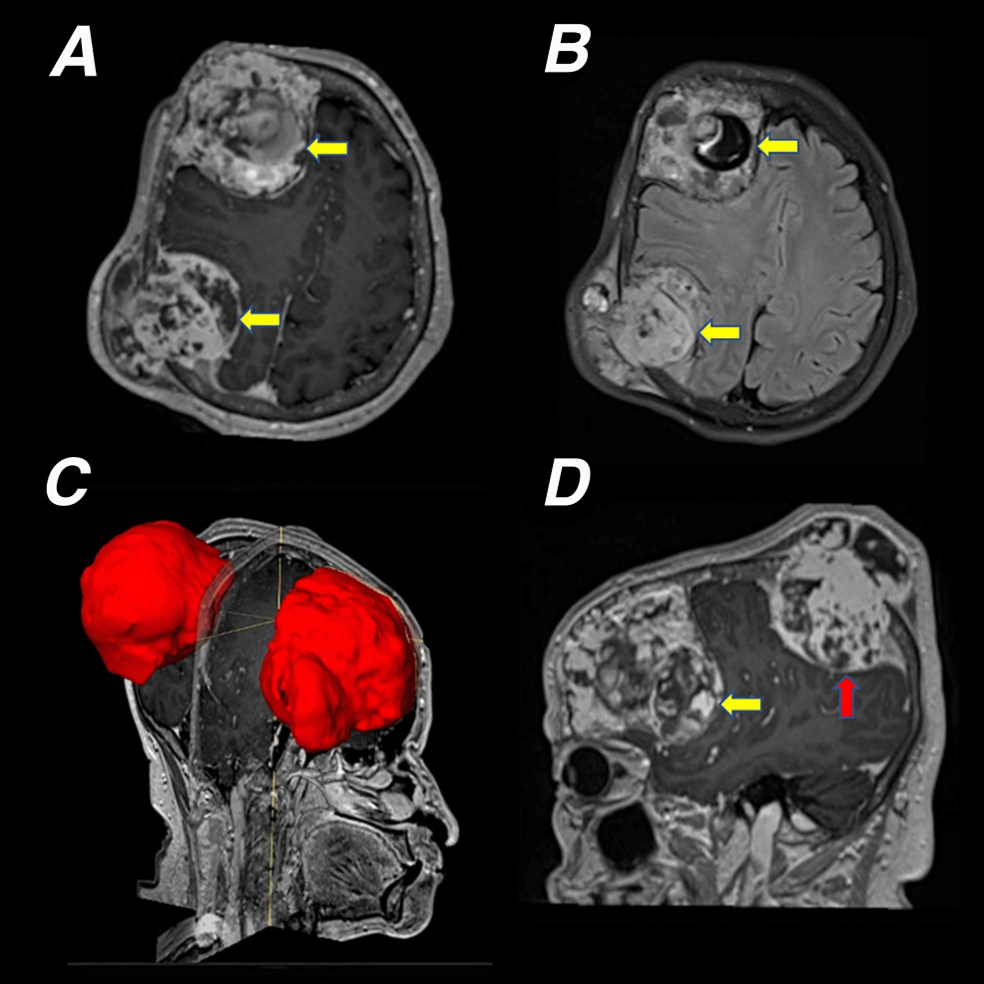
Brain magnetic resonance imaging. (A) Axial section in T1 sequence with right hemisphere lesions with bone and soft tissue invasion and intracranial and extracranial components; enhancement of the solid portions after intravenous contrast medium (yellow arrows). (B) An axial section in FLAIR sequence shows two multilobed and heterogeneous lesions with a hyperintense signal compatible with necrosis areas (yellow arrows). (C) Right sagittal section in 3D reconstruction sequence of both lesions and their relationship with the brain and skull. (D) The left sagittal section in T1 sequence with intravenous contrast medium shows the largest lesion in the frontal and temporal lobes with secondary compression of the lateral ventricle. Surrounding edema and displacement of midline structures up to 2 cm to the left (yellow arrow). The parietal lesion is compressing the parenchyma and displacing the basal nuclei and the mesencephalic peduncle (red arrow). FLAIR: fluid-attenuated inversion recovery; 3D: three-dimensional.

MRI scanned two bone lesions that were destroying and expanding the right skull bone with intracranial extension. Both were multilobulated, with cystic and solid components. They showed a hyperintense signal on T2 and FLAIR (fluid-attenuated inversion recovery), consistent with necrosis areas, and a hyperintense signal on T1 and SWI (susceptibility-weighted imaging), compatible with chronic hemorrhage. Enhancement of the solid components was observed after the administration of intravenous contrast media. The largest lesion in the frontal and temporal regions had two lobes and measured 8 cm × 7.7 cm × 7.8 cm, and it was compressing the frontal lobe and the lateral ventricle on the same side. The image also showed surrounding edema with a displacement of the midline brain structures up to 2 cm to the left. The parietal lesion measured 6.8 cm × 6.5 cm × 7.3 cm, and it was displacing the basal nuclei and the ipsilateral mesencephalic peduncle. The orbits, eyeballs, and maxillary sinuses were not affected (Figure 1).

Surgical approach

Surgical resection of both lesions was carried out in two separate procedures. The parietal lesion was approached first, followed by the frontal lesion nine days later. At the first surgery, a complete resection of the lesion was performed using a focused approach. A resection of the bone flap was made, followed by a methyl methacrylate-based cranioplasty. The blood loss during this first procedure was 1.2 liters. The macroscopic characteristics of the lesion during surgery were: bone invasion, high vascularization, multilobulated form, evident calcifications, apparent areas of necrosis, and hemorrhage.

**Figure 2:**
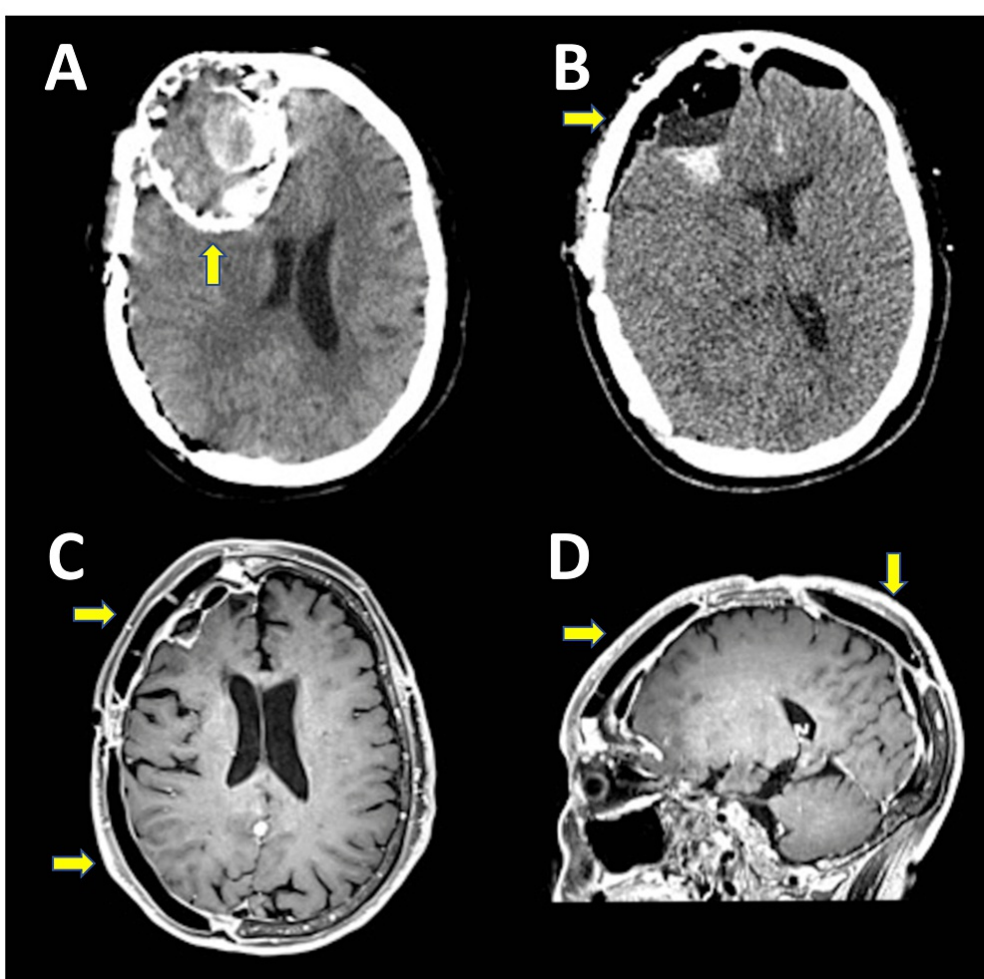
Preoperative and postoperative CT scans and MRI images. (A) An axial section in a CT scan shows a frontal tumor with soft tissue density and intracranial extension (yellow arrow). (B) An axial section with postoperative changes due to craniectomy and right frontal cranioplasty (yellow arrow). (C) An axial section in the MRI shows postoperative changes secondary to the resection of right frontal and parietal lesions (yellow arrows). (D) Postoperative MRI in a sagittal section showing (yellow arrows). CT: computed tomography.

The same was done for the frontal lesion. We observed a tumor with a bone capsule, highly vascular components, bone invasion, and necrotic areas inside the tumor. Blood loss during this second procedure was 300 ml (Figure 2).

Histopathology

**Figure 3:**
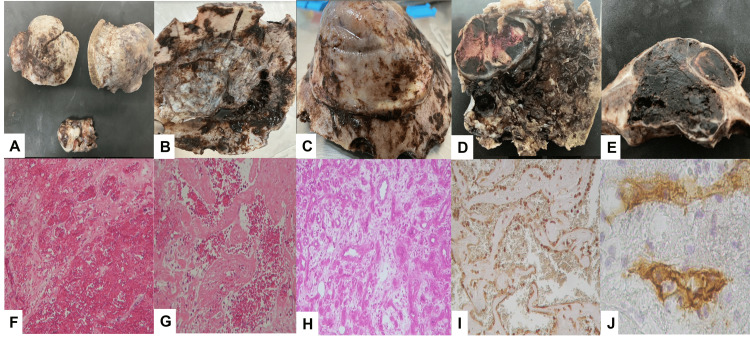
Gross and microscopic features of compound hemangioendothelioma with capillary and cavernous areas. (A–E) Right frontal and parietal lesions showed bone fragments. (F,G) Histologically, extensive necrosis involving more than 90% of the lesion is observed. It was formed by capillaries, blood lakes, and fibrin. No atypia or mitotic figures were observed. (H) The bone showed numerous vessels with an epithelioid pattern. (I) Immunohistochemical staining for vimentin shows cuboidal endothelial cells lining the vessels. (J) CD34 was positive on endothelial cells alone. CD: cluster of differentiation.

In the histological sections, bone was found with osteolytic and osteoblastic changes in the bone and fibroconnective tissue, and blood capillaries, capillary tubules, and small and large lumens formed blood lakes. Endothelial cells do not show cellular atypia or mitotic figures. In the most distal portion, there was extensive coagulative necrosis; right parietal extra-axial intraosseous lesions with a histopathological report of mixed hemangioendothelioma and extensive necrosis; calvarial bone with intraosseous hemangioendothelioma and extensive necrosis with desmoplastic changes; intracranial and extracranial right frontal intraosseous lesion with epithelioid hemangioendothelioma histopathology. Cells were positive for cluster of differentiation (CD) 34, CD31, factor VIII (FVIII), vascular endothelial growth factor (VEGF), and platelet-derived growth factor (PDGF); they were negative for cytokeratins, epithelial membrane antigen (EMA), vimentin, and P53. The Ki67 was 1% (Figure 3).

Follow-up

Follow-up was carried out by the neurosurgery, radiosurgery, and neuro-oncology services two months later. Thalidomide was prescribed as an adjuvant treatment, along with radiosurgery. The patient presented with significant improvement in left-side strength and no signs of cranial nerve involvement. She reported no more fluctuations in mental functions. The controlled imaging studies did not show tumor progression.

## Discussion

Clinically, HE shows malignant behaviors, such as invasion, recurrence, and metastasis. This tumor is considered a borderline brain tumor with a proliferative histopathological appearance and intermediate biological behavior between a hemangioma and an angiosarcoma [6,8,9].

HE has been described in adults ranging in age from 20 to 74 years. In children, the age reported is between 2 months and 15 years. The ratio between men and women is about 1.2:1 in adults and 3.5:1 in children [1,5]. It is more common to find extra-axial localization in 62-67% of the cases [5].

The symptoms associated with these tumors depend mainly on their location. They can be related to headaches, seizures, and limb weakness. Within the extracranial location, it is more frequently found in the liver, lungs, or skin. It is important to scan the chest and abdomen because of the possibility of metastasis [10-12].

MRI data in T1 and T2 sequences is consistent with isointense, hyperintense, or heterogeneous signal lesions. In half of the cases, they present perilesional edema [5]. Other characteristics are flow voids in the T2 sequence, heterogeneous enhancement with the administration of intravenously administered contrast medium, and hemorrhage data in 25% of the patients [13-20]. Other studies can be used, such as angiography, positron emission tomography/computed tomography (PET/CT), or bone scintigraphy, to identify tumor vasculature supplied mainly by the middle meningeal artery, external carotid artery, anterior cerebral artery, and internal carotid artery. In the case of bone invasion, the main thing is to observe osteolytic changes, bone shells, and honeycomb configurations [21-22].

Within the treatment modalities, surgery is included as the first choice because it is associated with a favorable prognosis without the need for adjuvant therapy. However, when it is required, it is recommended to continue with radiotherapy and chemotherapy [4,9,23,24]. The fact that this type of tumor has a mostly intracranial location makes it difficult to perform a complete resection in 60% of cases because of the invasion of bone, meninges, muscle, arteries, and soft tissues, resulting in a high rate of intraoperative bleeding, abandonment of the procedure, and its association with complications in almost 16% of cases. This is why some authors recommend preoperative embolization to reduce the risk of intraoperative bleeding [4,6,15,17,22,25]. Kunzita et al. [6] attempted to perform a resection of a huge tentorial HE, but the surgery was stopped due to unexpectedly profuse bleeding. After embolizing the tumor, they achieved a successful resection. In our case, the hospital did not have the necessary materials to perform this procedure, so a total resection was scheduled from the start. With adequate hemodynamic control during surgery, bleeding was not a limiting factor for the successful extraction of the two tumors. Adjuvant treatment modalities include radiotherapy associated with surgery as well as chemotherapy with interferon or lenalidomide in the long term, particularly in cases where there are metastases or multiple lesions [20,22,23].

The prognosis for this type of tumor and its associated intracranial locations is not well established due to its rare presentation and the limited series of cases that exist. The recurrence rate corresponds to 20-24%, and regarding the rate of metastasis, it is estimated that it can occur in 15-20% of all patients [4,17,26]. The mortality rate associated with these lesions is related to their high vascularity and corresponds to 12-15% [5]. That is why the proper collection of cases like this and their correct follow-up can help us create standardized criteria regarding initial management, proper classification, and selecting the best adjuvant management for HE.

## Conclusions

Adequate identification and approach selection are important since, although HE is a rare tumor with low proliferative activity, it is extremely invasive with a high risk of complications associated with its treatment. This is why a more extensive study must be carried out prior to surgery to rule out a metastatic process and to give a better follow-up due to the possibility of recurrence. Therefore, specific criteria must be established for its maximum resection with embolization support and, if necessary, subsequent adjuvant management with radiosurgery and chemotherapy.
